# Response to “Physical activity and psychological support can replace “another pill” to manage cancer-related symptoms in children and adolescents diagnosed with cancer”

**DOI:** 10.1186/s12906-024-04476-4

**Published:** 2024-04-22

**Authors:** Dana C. Mora, Miek C. Jong, Sara A. Quandt, Thomas A. Arcury, Agnete E. Kristoffersen, Trine Stub

**Affiliations:** 1 National Research Center in Complementary and Alternative Medicine, Department of Community Medicine, Faculty of Health Science, NAFKAM, UiT The Arctic University of Norway, Tromsø, 9037 Norway; 2 Department of Epidemiology and Prevention, Division of Public Health Sciences, Wake Forest University School of Medicine, Winston-Salem, NC 27157 USA; 3 Department of Family and Community Medicine, Wake Forest University School of Medicine, Winston-Salem, NC 27157 USA

Thank you for the opportunity to respond to the letter to the editor submitted by Dr. Caru and his colleagues [1]. We appreciate the interest of Dr. Caru et al. in our paper and their knowledgeable comments on our study.

In their letter to the editor [1], Dr. Caru notes that the findings of our study [2] need to be put into perspective to increase its scope and highlight that physical activity and psychosocial interventions are important nonpharmacological interventions to manage cancer-related symptoms. Furthermore, they point out that our paper [2] did not discuss physical activity and psychological support as CAM modalities. We agree that the latter were not discussed in the paper to a great extent. However, this reflects the use of a qualitative interview design in which we focused on modalities that the participants themselves discussed in response to the queries and probes used to illicit their views on supportive care for children undergoing cancer treatment. The interviews were open-ended, and, while participants’ responses were probed for additional modalities, such an approach does not query about specific modalities not discussed by the participants. Some participants briefly mentioned physical activity, but it was not spoken about in enough detail or by enough participants to emerge as a salient theme in the data analysis. We briefly discuss psychosocial support in the results section under the play, psychodrama, and music therapy Sect. [2].

As stated in the paper’s methods section, the participants were recruited using purposive sampling [3] through professional pediatrics and CAM networks. One of the limitations of the paper was that it was not possible to interview providers for every CAM modality currently used. We agree that our study had limitations and think it is important to highlight modalities (such as physical activity and psychosocial interventions) that are safe and effective and can aid parents and children in coping with the adverse effects derived from cancer treatment.

In their letter, Dr. Caru and colleagues appear to criticize our paper because it did not include experts on physical activity. This criticism misses the point that we interviewed care providers with expetice in treating pediatric oncology patients not necessarily researchers. We do understand that there have been studies published of the effectiveness of physical activity interventions with child cancer patients [4]. However, this does not necessarily make such interventions top of mind with care providers, the population we interviewed. It is indeed significant that such interventions were NOT noted by our study participants. We suggest that this reflects the need for greater training of care providers in the effectiveness of such modalities, rather than a shortcoming of our study.

In conclusion, we agree with Dr. Caru and his colleagues that physical activity and psychosocial interventions are important nonpharmacological interventions to manage cancer-related symptoms among children and youth with cancer. We are glad they took an interest in our work and expanded its scope, as this enriches the field of study and further highlights modalities that help lessen the burden children diagnosed with cancer and their families must endure.

## Data Availability

Not applicable.
